# Investigation of *Proteus vulgaris* and *Elizabethkingia meningoseptica* invasion on muscle oxidative stress and autophagy in Chinese soft-shelled turtle (*Pelodiscus sinensis*)

**DOI:** 10.1038/s41598-021-83388-6

**Published:** 2021-02-11

**Authors:** Hong-Hui Li, Ling-Sheng Bao, Shi-Ming Deng, Li Liu, Jia Cheng, Xiao Chen, Ya-Xiong Pan, Jian-She Zhang, Wu-Ying Chu

**Affiliations:** 1 Hunan Provincial Key Laboratory of Nutrition and Quality Control of Aquatic Animals, College of Biological and Environmental Engineering, Changsha University, Changsha, China; 2 College of Chemistry and Food Engineering, Changsha University of Science & Technology, Changsha, China; 3 Hunan Fisheries Science Institute, Changsha, China

**Keywords:** Biochemistry, Molecular biology, Physiology, Zoology

## Abstract

Muscle is an important structural tissue in aquatic animals and it is susceptible to bacterial and fungal infection, which could affect flesh quality and health. In this study, Chinese soft-shelled turtles were artificially infected with two pathogens, *Proteus vulgaris* and *Elizabethkingia meningoseptica* and the effects on muscle nutritional characteristics, oxidative stress and autophagy were assayed. Upon infection, the muscle nutritional composition and muscle fiber structure were notably influenced. Meanwhile, the mRNA expression of Nrf2 was down-regulated and Keap1 up-regulated, thus resulting in a decrease in antioxidant capacity and oxidative stress. However, with N-acetylcysteine treatment, the level of oxidative stress was decreased, accompanied by significant increases in antioxidant enzyme activities and the mRNA levels of SOD, CAT, GSTCD, and GSTO1. Interestingly, there was a significant increase in autophagy in the muscle tissue after the pathogen infection, but this increase could be reduced by N-acetylcysteine treatment. Our findings suggest that muscle nutritional characteristics were dramatically changed after pathogen infection, and oxidative stress and autophagy were induced by pathogen infection. However, N-acetylcysteine treatment could compromise the process perhaps by decreasing the ROS level and regulating Nrf2-antioxidant signaling pathways.

## Introduction

Muscle is not only an important structural tissue and locomotive organ but also serves as the largest protein repository in aquatic animals^1–3^. However, the muscle tissue of aquatic animals is susceptible to various pathogenic bacteria in the process of aquaculture, which affects the muscle quality and health, and thereby affecting the aquatic food safety and aquaculture development.

In recent years, several studies have shown that infected by pathogenic microorganisms could induce the production of large amounts of reactive oxygen species (ROS) within organisms^4^. The study of free radical biology has found that the pathogenic factors of many difficult diseases are directly related to the toxic reaction of excessive free radicals^5^. For example, *Pseudomonas aeruginosa* is a kind of opportunistic pathogen with a wide range of pathogenic factors, which is specific aerobic bacteria. It could be induced oxidative stress of host cells, release a large number of reactive oxygen species, and produce toxic reactions to organism^6,7^. The toxin inhibits phagocytosis in macrophages^7^ and causes the premature neutrophil senescence and apoptosis^8^. *P. aeruginosa* could be produced free radicals by inhibition endogenous antioxidants, glutathione (GSH) and catalase (CAT), and may utilize the direct oxidation of NADPH to produce free radicals^9,10^. Studies have shown that ROS resulting from oxidative stress also plays a significant role in the pathogenesis of *Helicobacter pylori*. In the study of *H. pylori* infection, the bacterial colonization was related to the increase of NO and malondialdehyde (MDA), and DNA damage in the gastric mucosa^11^. The increase of ROS or lack of antioxidants can cause the imbalance of antioxidant system, leading to a series of oxidative stress injuries, including lipid peroxidation, protein denaturation, DNA damage and enzyme activity alterations^12^. Meanwhile, the organism activating a series of defensive responses to prevent further oxidative damage by increasing the activity of antioxidant enzymes and activating lysosomal enzymatic degradation in vivo^13^. NF-E2-related factor 2 (Nrf2) is an important transcription factor regulating the antioxidant stress response of cells. It binds to the antioxidant response element (ARE), and then initiates the transcription of the target gene mediated by ARE to enhance the resistance of cells to oxidative stress^14^. The Kelch-like ECH-associated protein 1 (Keap1)-Nrf2-ARE signaling pathway is one of the most important cellular defense mechanisms against oxidative stress^15,16^. In addition, ROS also involved in a variety of signaling pathways to induce autophagy, and play an important role in the formation of autophagy^17^. When autophagy occurs, cells encapsulate degraded organelles or cytoplasm into vesicular autophagosomes through monolayer or bilayer membranes under the regulation of autophagy-related genes, and then the contents are degraded by a series of hydrolytic enzymes in lysosomes to achieve cell metabolism and energy renewal. Therefore, autophagy and oxidative stress are closely related, and they play important roles in cellular stress response, defense function and damage^18,19^.

N-acetylcysteine (NAC) as a precursor of glutathione, is an antioxidant containing thiol group, which has the functions of scavenging oxygen free radicals, regulating cell metabolism, preventing DNA damage, regulating gene expression and signal transduction system^20–22^. However, it has been shown that the antioxidant NAC can not only significantly reduce the level of ROS, but also decrease the cell death induced by AS and inhibit the formation of vacuoles^23,24^. In addition, NAC is widely used in the study of the role of ROS in many biological and pathological processes^22^.

Chinese soft-shelled turtle is a model animal of reptiles. Because of its excellent meat quality and high nutrition values, it was considered to be an ideal material for studying on nutritional quality of aquatic animals. Meanwhile, it has an important significance to improve protein and amino acid nutrition in humans. Furunculosis is a common disease with high incidence, rapid dissemination, and strong pathogenicity in the aquaculture of Chinese soft-shelled turtles. We found two furunculosis pathogens, *Proteus vulgaris* and *Elizabethkingia meningoseptica* threaten the turtles. *P. vulgaris* is an opportunistic pathogen with great potential harm to aquatic animal health and aquaculture development^25,26^. *E. meningoseptica,* a gram-negative rod widely distributed in fresh and saltwater, in the soil, and in some animals, is resistant to most β-lactam antibiotics^27^. At present, the effects and molecular mechanism of these two furunculosis pathogens on the muscle nutritional quality and health of turtles still remain largely unknown. In this study, we analyzed the effect of *P. vulgaris* and *E. meningoseptica* on muscle nutritional characteristics, oxidative stress and autophagy in turtles. Our study provides a theoretical basis for elucidating the damage caused by bacterial infection in aquatic animals, at least in Chinese soft-shelled turtles, and may put forward its potential significance in muscle nutritional quality, health, and disease control.

## Materials and methods

### Ethics and statements

This study was approved by the Research Ethics Committee of the Nutritional Quality and Health of Changsha University (Changsha, China), and all animal experiments have complied with the ARRIVE guidelines. Besides, all methods were performed following the relevant guidelines and regulations.

### Experimental animals and strains

Healthy juvenile turtles and furunculosis pathogens, namely Lb18-01 (*P. vulgaris*) and Lb18-02 (*E. meningoseptica*), provided by Hunan Fisheries Science Institute (Changsha, China). After one week of feeding, a total of 135 healthy turtles of similar size were randomly assigned to 3 groups that normal control group (NC), infection group (Lb18), and NAC-treated infection group (Lb18 + NAC) with 3 replicates per group (n = 3 replicate tanks), and 15 turtles in each replicate. The initial body weight was approximately 100 g. During the experimental period, water temperatures were kept at 28 to 30 °C, and the experiment was under the natural light cycle and they were fed basal diets.

### Artificial infection and drugs

A bacterial suspension was prepared from pathogenic bacteria that were isolated and identified. The bacterial suspensions (0.1 ml) were used to artificially infect healthy turtles via abdominal cavity injection (bacteria density, 1.0 × 10^8^ CFU/ml), and then NAC treatment of diseased turtles at 2 weeks after the infection. The antioxidant NAC (Sigma) was dissolved in 0.7% normal saline. The turtles assigned to the Lb18 + NAC group received an intraperitoneal injection of NAC for 2 weeks (150 mg/kg NAC every other day). The NC and Lb18 groups were injected with an equal volume of 0.7% normal saline.

### Sample collection and nutritional composition analysis

At the end of the experimental period, the turtles in each tank were weighed and measured. After anesthetized with MS-222 (Green Hengxing Biotech Co., Ltd., Beijing, China). The survival rate (SR), body weight and hepatosomatic index (HSI) were calculated based on the turtles’ total population and body weight. Blood samples were collected from the jugular veins and then centrifuged (3500 × g, 15 min) at 4 ℃, and the supernatant was collected and stored at -80 ℃ until analysis. White muscles of limb and other tissues (liver, intestinal, spleen, kidney) were collected and immediately placed into liquid nitrogen and stored at -80 ℃ for use. The amino acid content in the tested samples was determined with an automatic amino acid analyzer (Hitachi Model L8900). The moisture content was determined by the direct drying method. Place the sample in an oven at 105 ℃ and dry to constant weight to determine the moisture content. Protein content was determined by Kjeldahl method. Crude lipid content was determined by Soxhlet extraction with petroleum ether as solvent^28^.

### Serum biochemical indices analysis

The contents of total protein (TP), albumin (ALB), glucose (GLU), triacylglycerol (TG), and total cholesterol (TC) and the activities of aspartate aminotransferase (AST), alanine aminotransferase (ALT), and alkaline phosphatase (ALP) in the serum were determined using a kit (Nanjing Jiancheng Biological Engineering Research Institute of China).

### Histopathological analysis

The muscle tissue was collected, the thickness was not more than 0.5 cm, and put into 4% paraformaldehyde tissue fixative solution. Paraffin embedded, and the thickness of paraffin section was 5 μm. The histological paraffin section technology and hematoxylin eosin (HE) staining were used, and the histopathological observation was performed with Olympus BX53 microscope (400 ×).

### Antioxidant system-associated indicator assay

The muscle samples were rinsed with 0.70% saline, then homogenized on ice with 1:9 volume (v/w) saline. Then, the samples were centrifuged at 4000 rpm for 15 min at 4 ℃ respectively, and the supernatant was collected for determination. The content of total protein (TP) was determined by BCA method. The activity of total superoxide dismutase (T-SOD), glutathione peroxidase (GPx), glutathione S-transferase (GST), catalase (CAT), glutathione reductase (GR) and reduced glutathione (GSH), and the malondialdehyde (MDA), protein carbonyl (PC) contents were determined according to the results by the kit instructions of Nanjing Jiancheng Research Institute. The level of ROS was determined by a ELISA kit (ZciBio Technology Co., Ltd., Shanghai, China.) that were coated with the ROS antibody in 96 well plates, and the optical density (OD) of the reaction was measured at 450 nm by using enzyme-labeling instrument, and calculated sample concentrations^29^.

### Quantitative real-time PCR assay

Total RNA was extracted by Trizol method, and using the extracted RNA as a template, the first-strand cDNA was synthesized by reverse transcription kit according to the manufacturer’s protocol (Takara, Dalian, China). According to Li et al. (2019) studies, with the above cDNA as template and the quantitative real-time PCR reaction were performed for Nrf2, Keap1, SOD1 (CuZnSOD), SOD2 (MnSOD), CAT, GPx1, GPx2, GPx3, GPx4, GPx7, GSTCD, GSTK1, GSTO1, GSTP1, GSTZ1, GSR, ULK1, ULK2, ATG13, ATG101, Beclin-1, ATG14, MAP1LC3A, MAP1LC3B, MAP1LC3C, SQSTM1, AMPK, mTOR and β-actin (the housekeeping gene) by SYBR Premix Ex Taq II kit^30^. The genes primer sequence shown in Table 1. The relative expression of all detected genes was analyzed by 2^-ΔΔCt^ method^31^.

**Table 1:** Real-time PCR primer sequences.

Gene	Primer	length (bp)	Accession NO
Nrf2	F: 5′-TTCAAGCTGCCTTGATGCTC-3'	141	JX470526.1
	R: 5′-GCCTCACTGAACTGCTCCTTA-3'		
Keap1	F: 5′-GGGAGGTGGTCAAGCAGGAG-3'	119	XM_006129049.3
	R: 5′-GTCGATGCAGGCGTGGAA-3'		
SOD1 (CuZnSOD)	F: 5′-GCAGGTGCTCACTTCAATCC-3'	179	XM_006126060.3
	R: 5′-ACCACCATAGTGCGTCCAA-3'		
SOD2 (MnSOD)	F: 5′-CTTGCCTTATGACTATGGTGCC-3'	185	NM_001317049.1
	R: 5′-CATTGAACTTTAGGGCAGGCT-3'		
CAT	F: 5′-TGTGGGCAAACTTGTCTTGA-3'	168	NM_001286934.1
	R: 5′-GGTCCTAAACGGTGTCGGT-3'		
GPx1	F: 5′-CATCCTCCTCAGGCTTGTTATC-3'	108	XM_006117622.3
	R: 5′-ACATACAGGGCAAAGAGGTCAC-3'		
GPx2	F: 5′-GCACGGCACAAGCAGGTCTC-3'	182	XM_014571095.2
	R: 5′-GGCAGGTGGGCCTTCAGGTA-3'		
GPx3	F: 5′-GGGAACAATCTACAACTATGGG-3'	241	JX470527.1
	R: 5′-GCTTGCCAAACTGGTTGCTCG-3'		
GPx4	F: 5′-TCCAAATGAGGCAAGACG-3'	209	JX470528.1
	R: 5′-GCGTTGTTCCCGTTGACC-3'		
GPx7	F: 5′-CGGATTTACAGACAGCCACTA-3'	178	XM_006126395.3
	R: 5′-GCTGAACATAGGGAAGGAG-3'		
GSTCD	F: 5′-AAGAACGCTGCTGCCAAATGC-3'	211	XM_014571622.2
	R: 5′-TAGGGCAGGGATGAGGAGA-3'		
GSTK1	F: 5′-CACCCACCCAGACAGTATATTG 3'	114	XM_025184413.1
	R: 5′-TCCTTCAGGCGATTCTTCAC 3'		
GSTO1	F: 5′-TTTGGCTTGGTGCCTATTCTG-3'	148	XM_006135385.2
	R: 5′-CCAAGAGCATCCTTTGACACG-3'		
GSTP1	F: 5′-GTAACCCTGTACCAGTCCAACG-3'	167	XM_025182286.1
	R: 5′-CCCTGGCTCACATAGTTCTGGT-3'		
GSTZ1	F: 5′-TGGCATCCTCCTGTCTCAAT-3'	143	XM_006134809.3
	R: 5′-AGGGCTGAATGCCAGAAACA-3'		
GSR	F: 5′-ACGTTGACTGTCTGCTGTGG-3'	194	XM_006125372.2
	R: 5′-TGCAACTGGAGTCAGGAGTG-3'		
ULK1	F: 5′-AGTTCCCAGAATCTACTCACA-3'	157	XM_014572209.1
	R: 5′-GCGACCTGCCCTTTATCT-3'		
ULK2	F: 5′-GAGCGATTTCATGGTGTGTGG-3'	162	XM_006138679.2
	R: 5′-ATAGGAGCCGTTTCACTGCGT-3'		
ATG13	F: 5′-GCACCATCACTCTGTCTTGT-3'	200	XM_006117292.1
	R: 5′-GTCTTCCACAGATGGGTATG-3'		
ATG101	F: 5′-TGGTGAACCTGGTCAGCGAGCA-3'	135	XM_006131161.3
	R: 5′-CCGACTGGGTGGGCATTTT-3'		
Beclin-1	F: 5′-AAAATCAGGGCAGAAGCAGAG-3'	144	XM_025185955.1
	R: 5′-CAACTGGACCTGGGCATAGCG-3'		
ATG14	F: 5′-CAAGGACCAGATACGGAGCAT-3'	163	XM_014572030.1
	R: 5′-ACTTCCGCCTGCTAAGGTTCT-3'		
MAP1LC3A	F: 5′-GAGCAGCATCCAAGCAAAAT-3'	102	XM_025188656.1
	R: 5′-CACGTGGTCAGGGACTAGAAAT-3'		
MAP1LC3B	F: 5′-GTAGAAGATGTGCGACTGAT-3'	193	XM_025178617.1
	R: 5′-AGAAGAAAGCCTGAGTGGAA-3'		
MAP1LC3C	F: 5′-TATGACTCTGACGCTGGCTGAA-3'	100	XM_006117436.3
	R: 5′-CACGAAACATCCAAACATCTCC-3'		
SQSTM1	F: 5′-GAGGCAGTTCCACTTGTCAGA-3'	211	XM_006138217.2
	R: 5′-GATAAGTGGGTCCAGTCCTCA-3'		
AMPK	F: 5′-GCAGGACCTCCCCAAGTATC-3'	164	XM_006139488.3
	R: 5′-TAGGCAACTGCTAAGGGGTC-3'		
mTOR	F: 5′-GTGGGAGGATGCCCTTGTAG-3'	173	XM_006127040
	R: 5′-CCATTTTGGCTTGCGTTTCA-3'		
β-actin	F: 5′-TGATGGACTCAGGTGACGGTGT-3'	158	XM_006112915.3
	R: 5′-GGCTGTGGTGGTGAAGCTGTAG-3'		

Nrf2, NF-E2-related factor 2; Keap1, Kelch-like-ECH-associated protein 1; SOD1 (CuZnSOD), copper, zinc superoxide dismutase; SOD2 (MnSOD), manganese superoxide dismutase; CAT, catalase; GPx, glutathione peroxidase; GSTCD, glutathione S-transferase C-terminal domain containing; GSTK1, glutathione S-transferase kappa 1; GSTO1, glutathione S-transferase omega 1; GSTP1, glutathione S-transferase pi 1; GSTZ1, glutathione S-transferase zeta 1; GSR, glutathione-disulfide reductase; ULK, unc-51 like autophagy activating kinase; ATG, autophagy related gene; Beclin-1, BECN1; MAP1LC3, microtubule-associated protein 1 light chain 3, SQSTM1, sequestosome 1; AMPK, AMP-activated protein kinase; mTOR, mammalian target of rapamycin.

### Western blot analysis

According to the experimental methods of Li et al. (2020), the muscle tissue was weighed (0.1 g) and 1 ml of lysis solution (50 mM Tris–HCl, 150 mM NaCl, 1 mM EDTA-Na2, 1% Triton X-100, 1% sodium deoxycholate and 0.1% SDS) was added for complete protein lysis. The tissue pieces were then homogenized and centrifuged for 10 min at 4 ℃ and 12,000 rpm. The content of TP of the supernatant was quantified by BCA method (Vazyme Biotech Co., Ltd, Nanjing, China). The protein samples were separated by SDS-PAGE and transferred to 0.45 μm PVDF membranes for analysis by Western blot. The membrane was sealed at room temperature for 1–2 h, and then incubated with primary antibody at 4 ℃ overnight. We used the anti-LC3A/B antibody (ab128025) at a 1/1000 dilution to probe the samples. Anti-β-actin (ab8224) at a 1/1000 dilution was used as a loading control. Western blots were scanned, and the bands were analyzed quantitatively by using NIH ImageJ software^32^.

### Transmission electron microscopy observation

The samples (2 mm × 2 mm × 2 mm) were fixed with 2.5% glutaraldehyde and then fixed with osmium tetroxide. The ultrathin sections were stained with uranyl acetate and lead citrate, and then observed by transmission electron microscopy.

### Statistical analysis

The statistical analysis was conducted using SPSS 18.0 software (SPSS, Chicago, IL, USA). All data were analyzed by (ANOVA) and then by Duncan's multiple range tests. The results were presented as mean ± standard error (S.E.), *p* < 0.05 was considered significant.

## Results

### Growth performance and serum biochemical indices

The survival rate and body weight were significantly affected after infection, and Lb18 group was more apparent than NC and Lb18 + NAC groups (*p* < 0.05). The HSI of Lb18 and Lb18 + NAC group were both significantly lower than that of NC group (*p* < 0.05), but no significant difference was observed between Lb18 + NAC and Lb18 (*p* > 0.05) (Table 2). As shown in Table 3, the contents of TP, ALB, and GLU in the serum of Lb18 and Lb18 + NAC groups were significantly lower than those in NC group (*p* < 0.05), while the contents of TP and ALB in Lb18 + NAC group were slightly higher than that in Lb18 group without significant difference (*p* > 0.05). However, the AST, ALT, ALP, TG, and TC levels in Lb18 and Lb18 + NAC groups were significantly higher than those in NC group (*p* < 0.05). The Lb18 + NAC group had significantly decreased AST, ALT, TG, and TC levels compared to Lb18 group (*p* < 0.05).

**Table 2:** Effects of infection with *P. vulgaris* and *E. meningoseptica* on growth performance of Chinese soft-shelled turtles.

	NC	Lb18	Lb18 + NAC
Initial body weight (g)	99.83 ± 1.78	100.06 ± 1.96	99.94 ± 1.77
Final body weight (g)	124.11 ± 2.43^c^	95.96 ± 2.96^a^	105.73 ± 2.28^b^
HSI (%)	3.36 ± 0.18^b^	3.04 ± 0.16^a^	2.94 ± 0.13^a^
SR (%)	100 ± 0.00^b^	88.89 ± 0.04^a^	95.56 ± 0.05^b^

Values represent the mean ± S.E. (n = 3 replicate tanks), all turtles were sampled for each tank. Values within the same row having different superscripts are significantly different (*p* < 0.05).

**Table 3:** Effects of physiological and biochemical characteristics of serum in each group.

	NC	Lb18	Lb18 + NAC
TP (g/L)	28.43 ± 0.55^b^	18.01 ± 1.21^a^	20.07 ± 1.41^a^
ALB (g/L)	13.72 ± 0.90^b^	8.22 ± 0.74^a^	9.29 ± 0.69^a^
AST (U/L)	158.59 ± 3.01^a^	425.31 ± 5.32^c^	352.59 ± 6.51^b^
ALT (U/L)	6.49 ± 0.81^a^	69.52 ± 1.99^c^	53.91 ± 1.00^b^
ALP (U/L)	34.22 ± 1.71^a^	43.66 ± 2.33^b^	42.09 ± 1.57^b^
GLU (mmol/L)	9.31 ± 0.68^b^	0.66 ± 0.09^a^	0.63 ± 0.06^a^
TG (mmol/L)	4.61 ± 0.41^a^	15.43 ± 0.99^c^	8.58 ± 1.01^b^
TC (mmol/L)	6.29 ± 0.57^a^	11.69 ± 1.18^b^	8.21 ± 1.09^a^

TP, total protein; ALB, albumin; AST, aspartate aminotransferase; ALT, alanine aminotransferase; ALP, alkaline phosphate; GLU, glucose; TG, triglyceride; TC, total cholesterol.

Values represent the mean ± S.E. (n = 3 replicate tanks), 3 turtles were sampled for each tank. Values within the same row having different superscripts are significantly different (*p* < 0.05).

### Changes in muscle nutritional composition and histopathology

**Figure 1:**
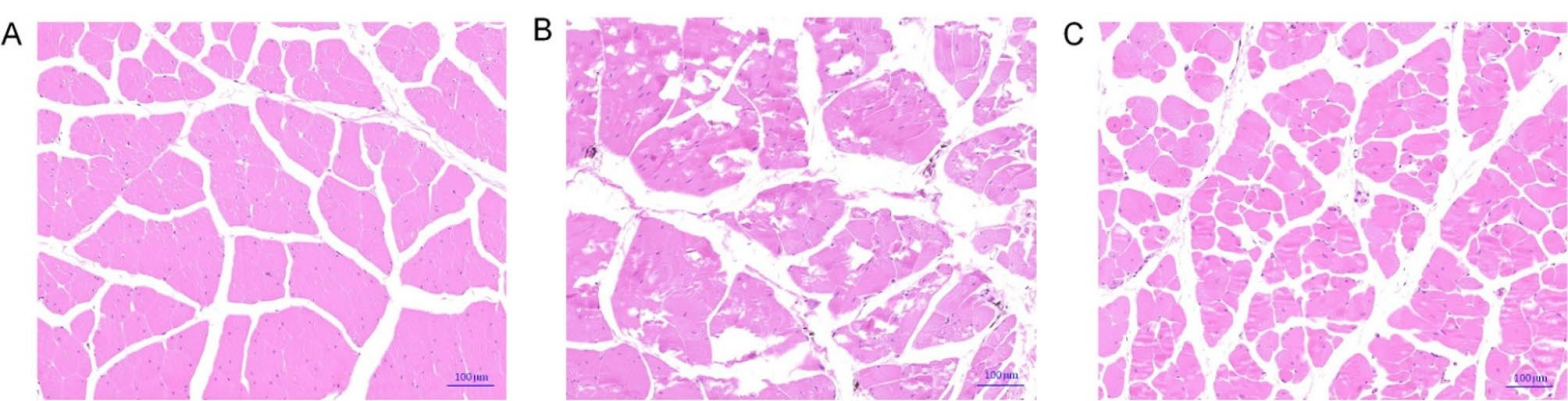
Histopathological observation of Chinese soft-shelled turtles infected with furunculosis pathogens. A was normal control group (NC), B was infection group (Lb18), C was infection treated with NAC group (Lb18 + NAC).

The muscle crude protein content was decreased, and the moisture and lipid contents were increased in Lb18 group compared with NC group (*p* < 0.05). However, the crude protein content was obviously increased, the lipid content was decreased in Lb18 + NAC group compared with Lb18 group (*p* < 0.05), and moisture was not significantly different (*p* > 0.05) (Table 4). Similarly, the content of amino acids in the muscle of Lb18 group was lower than that of the other groups, and the change in different kinds of each amino acid was different. The Lb18 + NAC group was increased slightly, but there was no significant difference (*p* > 0.05) and it was still lower than that of NC group (Table 5). HE staining results showed that muscle fibers became widen interspace, necrotic just like wax and even dissolved in Lb18 group. The degree of muscle fiber, cellular, and stromal swelling was improved in Lb18 + NAC group compared with Lb18 group (Fig. 1).

**Table 4:** Effects of infection with *P. vulgaris* and *E. meningoseptica* on muscle composition of Chinese soft-shelled turtles.

	NC	Lb18	Lb18 + NAC
Moisture (%)	77.63 ± 1.05^a^	82.19 ± 1.91^b^	81.58 ± 1.60^b^
Crude protein (%)	19.37 ± 1.18^c^	16.09 ± 1.22^a^	17.95 ± 1.33^b^
Crude lipid (%)	1.02 ± 0.27^a^	1.65 ± 0.59^c^	1.37 ± 0.56^b^

Values represent the mean ± S.E. (n = 3 replicate tanks), 3 turtles were sampled for each tank. Values within the same row having different superscripts are significantly different (*p* < 0.05).

**Table 5:** Effects of infection with *P. vulgaris* and *E. meningoseptica* on amino acids composition analysis of muscle.

	NC	Lb18	Lb18 + NAC
Asp	1.67 ± 0.11^b^	1.35 ± 0.04^a^	1.45 ± 0.05^ab^
Thr	0.78 ± 0.05^b^	0.62 ± 0.03^a^	0.66 ± 0.02^a^
Ser	0.73 ± 0.05^a^	0.62 ± 0.01^a^	0.63 ± 0.02^a^
Glu	2.68 ± 0.18^b^	2.15 ± 0.04^a^	2.29 ± 0.08^ab^
Gly	0.80 ± 0.01^a^	0.81 ± 0.04^a^	0.74 ± 0.03^a^
Ala	0.97 ± 0.05^b^	0.83 ± 0.01^a^	0.84 ± 0.03^a^
Cys	0.05 ± 0.01^a^	0.04 ± 0.02^a^	0.07 ± 0.01^a^
Val	0.82 ± 0.06^a^	0.69 ± 0.04^a^	0.72 ± 0.03^a^
Met	0.47 ± 0.03^b^	0.37 ± 0.01^a^	0.39 ± 0.01^a^
Ile	0.84 ± 0.06^b^	0.67 ± 0.01^a^	0.71 ± 0.02^ab^
Leu	1.40 ± 0.09^b^	1.13 ± 0.02^a^	1.19 ± 0.04^a^
Tyr	0.62 ± 0.04^b^	0.49 ± 0.03^a^	0.53 ± 0.02^ab^
Phe	0.72 ± 0.05^b^	0.59 ± 0.02^a^	0.61 ± 0.02^a^
His	0.59 ± 0.00^a^	0.58 ± 0.05^a^	0.48 ± 0.04^a^
Lys	1.60 ± 0.10^b^	1.28 ± 0.02^a^	1.36 ± 0.04^a^
Arg	1.08 ± 0.06^b^	0.89 ± 0.02^a^	0.92 ± 0.03^ab^
Pro	0.57 ± 0.07^a^	0.60 ± 0.01^a^	0.59 ± 0.02^a^
**EAA**	**6.63 ± 0.41**^**b**^	**5.35 ± 0.14**^**a**^	**5.64 ± 0.19**^**a**^
**NEAA**	**9.76 ± 0.43**^**b**^	**8.36 ± 0.15**^**a**^	**8.54 ± 0.31**^**a**^
**TAA**	**16.39 ± 0.84**^**b**^	**13.71 ± 0.28**^**a**^	**14.18 ± 0.49**^**ab**^

EAA, essential amino acid (Thr, Val, Met, Ile, Leu, Phe, Lys); NEAA, nonessential amino acid (Asp, Ser, Glu, Gly, Ala, Cys, Tyr, His, Arg, Pro); TAA, total amino acid.

Values represent the mean ± S.E. (n = 3 replicate tanks), 3 turtles were sampled for each tank. Values within the same row having different superscripts are significantly different (*p* < 0.05).

### Antioxidant enzyme activities and gene expression patterns

**Figure 2:**
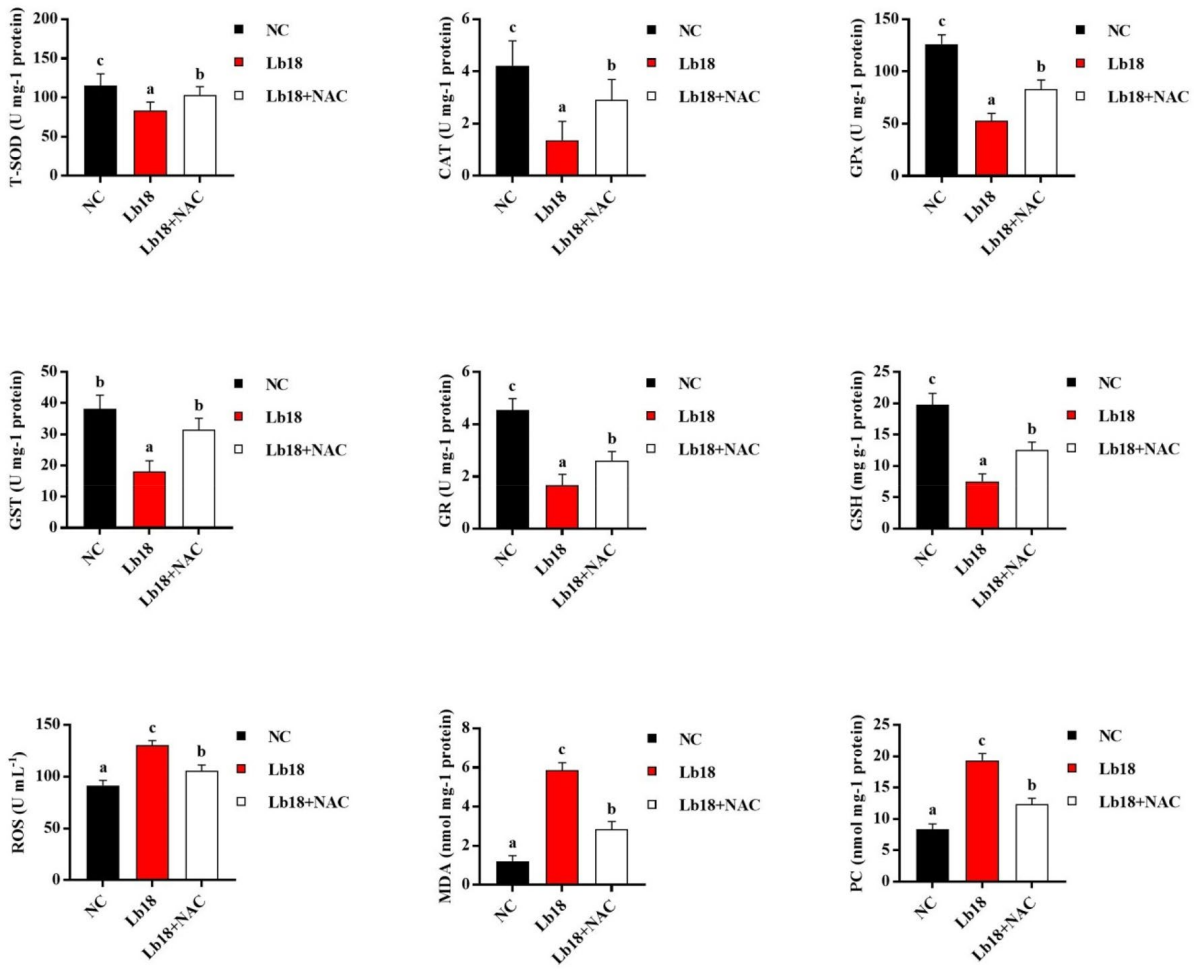
Effects of infection with *P. vulgaris* and *E. meningoseptica* on antioxidant system-associated indicators in the muscle of Chinese soft-shelled turtles. Values represent the mean ± S.E. (n = 3 replicate tanks), 3 turtles were sampled for each tank, the data with different letters show significant difference (*p* < 0.05). T-SOD, total superoxide dismutase; CAT, catalase; GPx, glutathione peroxidase; GST, glutathione-S-transferase; GR, glutathione reductase; GSH, glutathione. ROS, reactive oxygen species; MDA, malonaldehyde; PC, protein carbonyl.

**Figure 3:**
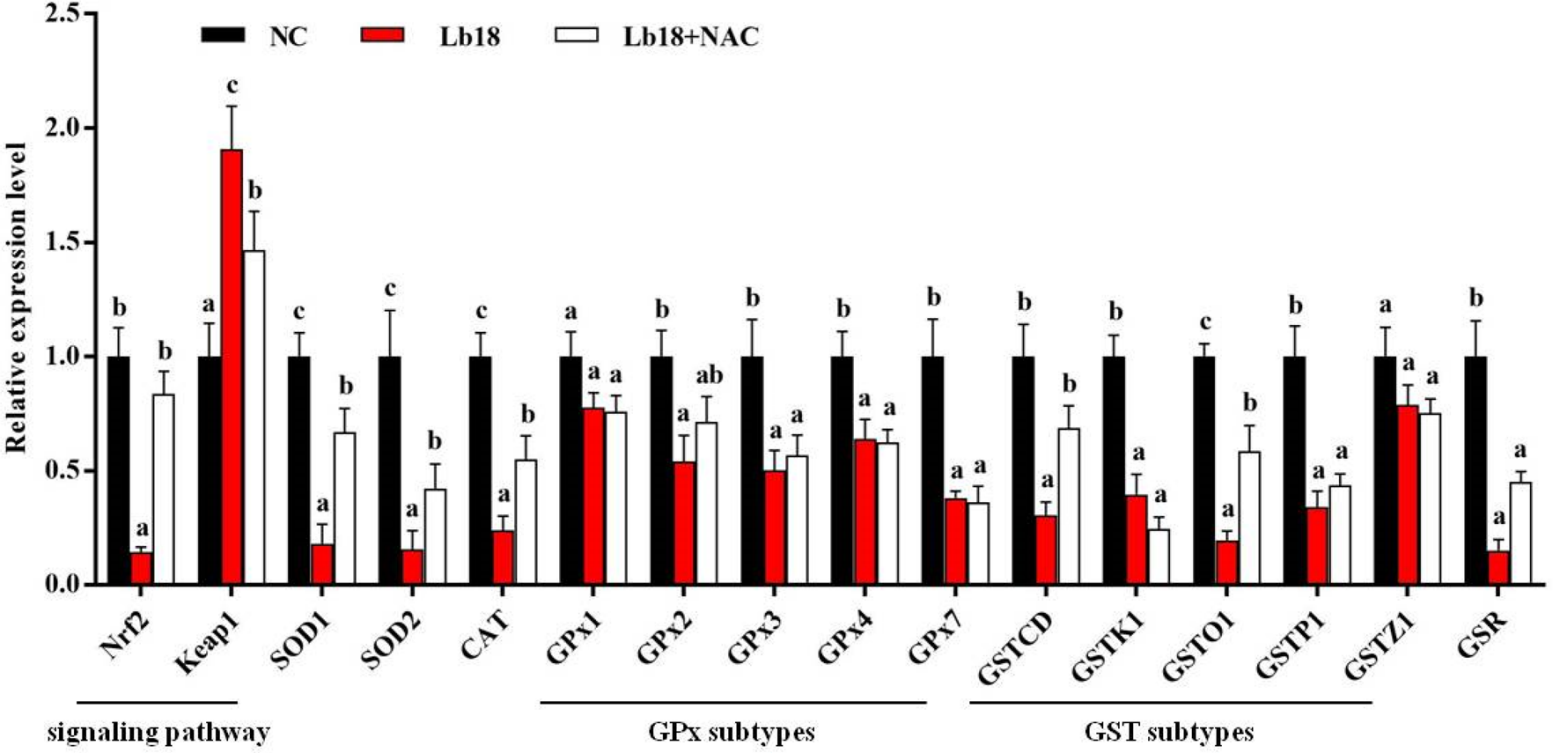
Relative expression of Nrf2 and antioxidant enzyme genes in the muscle of different groups. Values represent the mean ± S.E. (n = 3 replicate tanks), 3 turtles were sampled for each tank, the data with different letters show significant difference (*p* < 0.05).

The antioxidant enzyme activities of T-SOD, CAT, GPx, GST, GR and the content of GSH were significantly decreased in muscle tissues after infection with *P. vulgaris* and *E. meningoseptica* (*p* < 0.05). However, the contents of ROS, MDA and PC were significantly increased, which suggested that oxidative damage was accelerated in the turtles upon pathogen infection (*p* < 0.05). In contrast, the levels of ROS, MDA and PC in the infected turtles were significantly decreased by treatment with NAC, and the muscle antioxidant enzyme activities (T-SOD, CAT, GPx, GST, and GR) and the content of GSH were significantly increased in the muscle (*p* < 0.05) (Fig. 2). As shown in Fig. 3, the mRNA level of Nrf2 was significantly down-regulated as well as up-regulating Keap1 of muscle in Lb18 group (*p* < 0.05), while Nrf2 mRNA returned to normal levels and Keap1 was decreased in Lb18 + NAC group compared to Lb18 group (*p* < 0.05). Moreover, the expression of antioxidant genes (except GPx1 and GSTZ1) declined in Lb18 group (*p* < 0.05), but the expression of SOD1, SOD2, CAT, GSTCD, and GSTO1 in infected turtles increased significantly after treatment with NAC (*p* < 0.05).

### Changes in autophagy level

**Figure 4:**
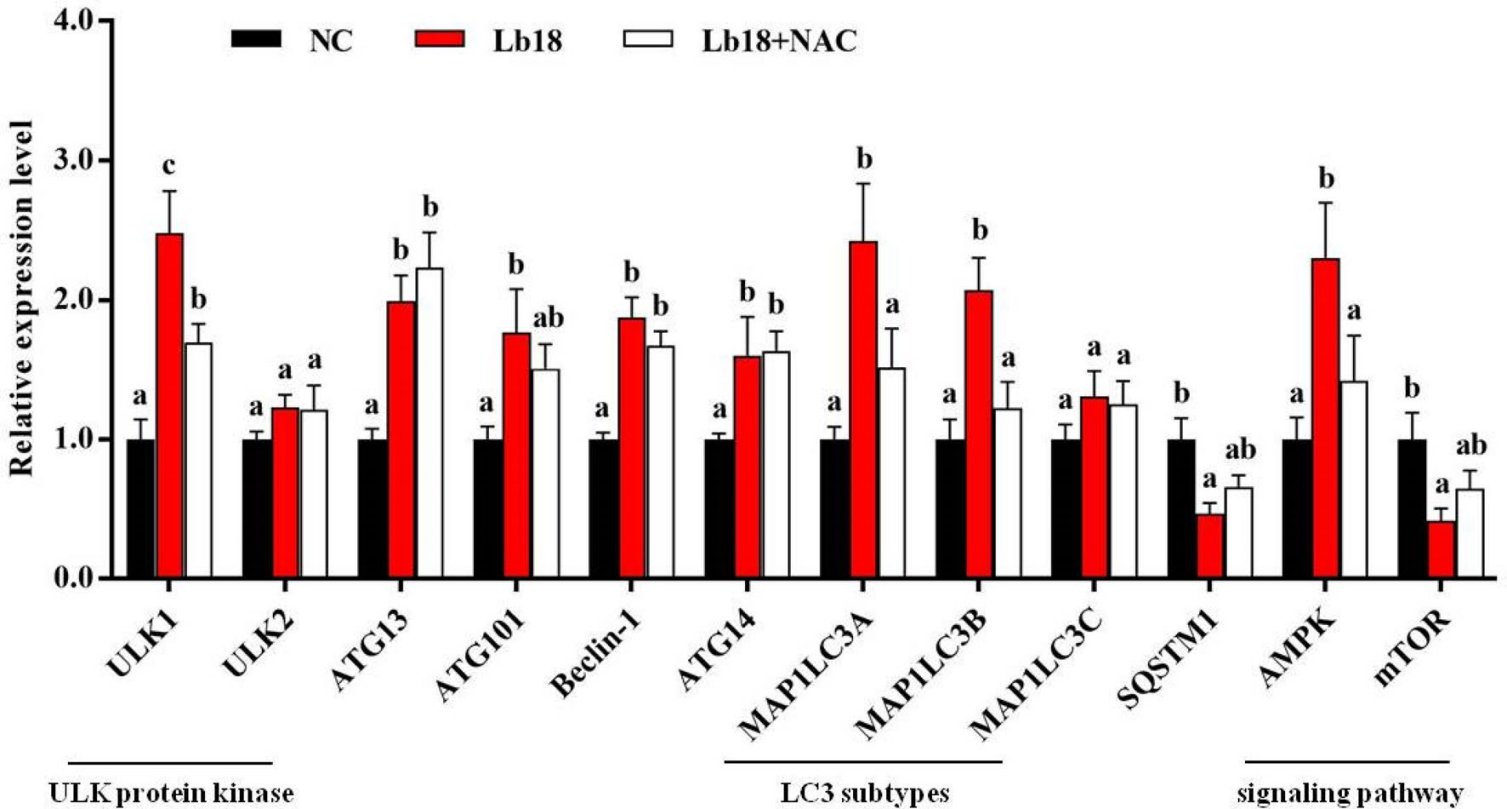
Relative expression of autophagy-related genes and AMPK-mTOR signaling pathway in the muscle of different groups. Values represent the mean ± S.E. (n = 3 replicate tanks), 3 turtles were sampled for each tank, the data with different letters show significant difference (*p* < 0.05).

**Figure 5:**
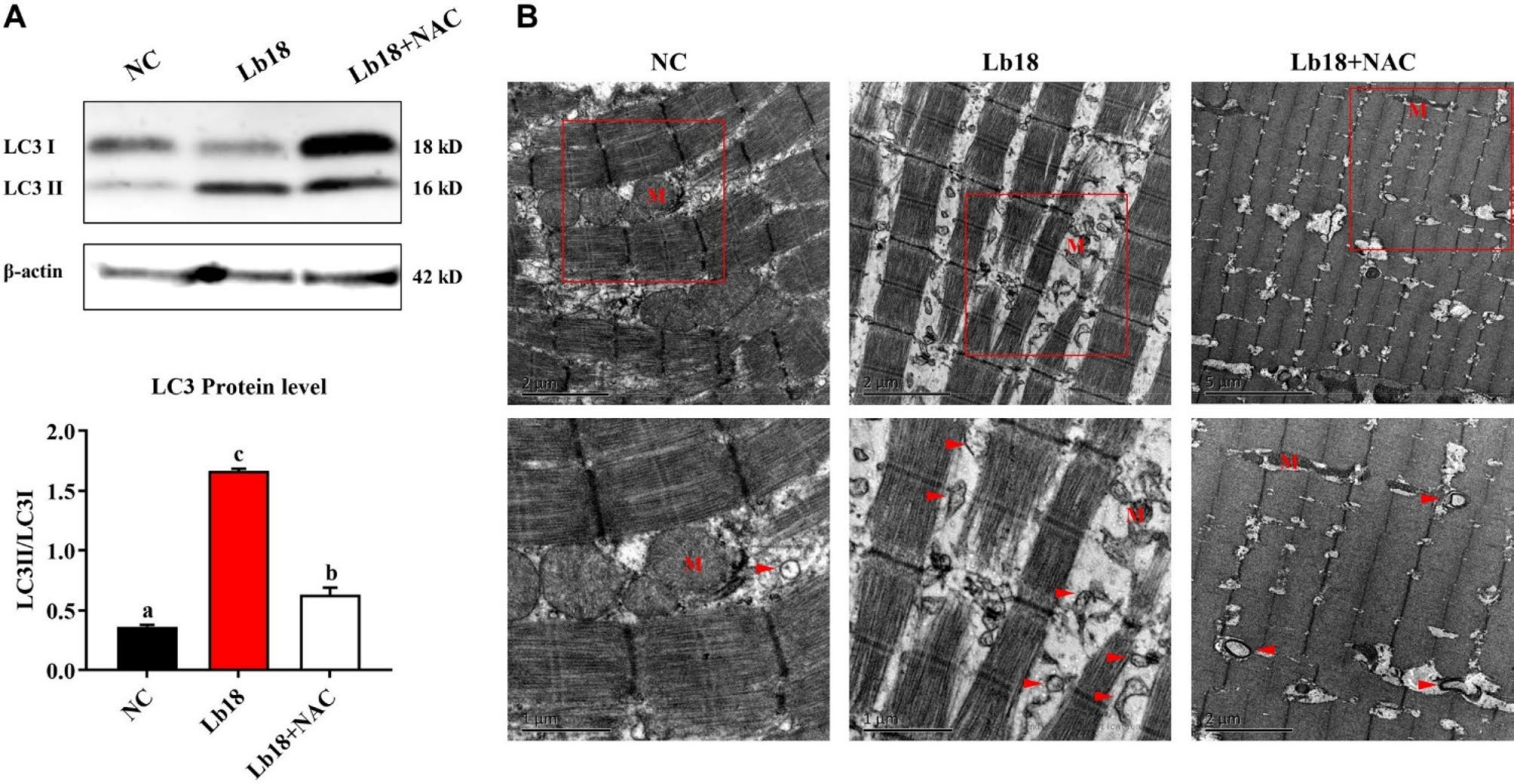
Autophagy detection using Western blot and transmission electron microscopy. (**A**) Western blot analysis of LC3 protein, and the bands were analyzed quantitatively by using NIH ImageJ software. (**B**) Autophagosome was observed by transmission electron microscopy. Red arrow heads indicate autophagosome, the letter M indicate mitochondrion.

The expression levels of the autophagy-related genes ULK1, ATG13, ATG101, Beclin-1, ATG14, MAP1LC3A, and MAP1LC3B were significantly increased, and SQSTM1 was decreased in Lb18 group (*p* < 0.05), while ULK1, MAP1LC3A and MAP1LC3B were significantly increased in Lb18 + NAC group compared to Lb18 group (*p* < 0.05). However, there was no significant difference in the other genes. In addition, significantly decreased mTOR and increased AMPK mRNA levels were observed in Lb18 group (*p* < 0.05), and AMPK returned to normal levels in Lb18 + NAC group (*p* < 0.05) (Fig. 4). Western blotting analysis showed that the expression of LC3 in Lb18 group was significantly increased, while Lb18 + NAC group was reduced significantly compared to that in Lb18 group but did not return to normal levels (*p* < 0.05) (Fig. 5A). Furthermore, TEM showed that there were obviously more autophagosomes in muscle with infectious pathogens than in those treated with NAC (Fig. 5B).

## Discussion

In this study, we found that the final body weight, HSI, and SR of turtles were significantly decreased after infected with *P. vulgaris* and *E. meningoseptica* (Table 2). The contents of serum TP, ALB and GLU were significantly decreased, and increased the contents of AST, ALT, ALP, TG and TC. After injection of NAC into the infected turtles, the AST, ALT, TG and TC levels were decreased (Table 3). These observations indicated that the growth performance and serum biochemical indices of turtles were affected by *P. vulgaris* and *E. meningoseptica* infection, and then affecting their immune function. However, treatment with NAC effectively alleviated the negative effects of pathogens infection on serum biochemical parameters of turtles, which may be related to its antioxidant effect and the improvement of immune function. This is similar to the results obtained in *Oreochromis niloticus*^33^, *Oncorhynchus mykiss* and *Salmo salar*^34,35^. The results of amino acid analysis showed that the content of amino acids in muscle of Lb18 infected group was lower than that of other groups, and the change in each amino acid was different. There was a slight increase in the Lb18 + NAC group, but it was still lower than NC group (Table 5). These results may be due to metabolic disorder in vivo after pathogenic bacterial invasion, destroying the original dynamic balance of amino acids and changing the amino acid pattern in muscle. It is generally accepted that the host experiences significant metabolic alterations after pathogen infection^36–38^, and slight changes in the metabolism of the infection site will significantly affect the outcome of the infection. Infection with *C. neoformans* perturbed the cysteine content in lung epithelial cell line after 6 h^39^. *Plasmodium yoelii* infection altered the amino acids content in plasma of the infected mice, including an increase in 10 amino acids and a decrease in 5 amino acids^40^. Similarly, *Plasmodium yoelii* infected affects the abundance of the amino acids content in red blood cells^41^. Subsequently, we found that muscle fibers were widening interspace, necrotic just like wax and even dissolved in the Lb18 group by HE staining, and the degree of muscle fiber, cellular and stromal swelling were improved in the Lb18 + NAC group compared with the Lb18 group (Fig. 1). These results indicated that the two pathogens had strong pathogenicity to turtles, and caused different degrees of pathological changes in muscle tissue. The degree of muscle injury was improved after NAC treatment. This is probably due to NAC helps improve the degree of muscle injury by reducing oxidative stress levels. There are similar reports in related studies, such as Takhtfooladi et al. (2014) reported that N-acetylcysteine could significantly reduce myocardial injury caused by ischemia–reperfusion in the skeletal muscle^42^. Zaki et al. (2017) reported marked improvement in an NAC-pretreated group when the myocardial cells of ISP treated group were disintegrated and destroyed by nucleolysis^43^.

Then, we further investigated the effect of pathogens infection on muscle antioxidant capacity of turtles, and the results showed that the antioxidant enzyme activity was decreased significantly in muscle after infected with *P. vulgaris* and *E. meningoseptica*. While the level of ROS, MDA and PC increased significantly in the muscle, and indicated that the oxidative damage accelerated in the turtles (Fig. 2). This may be due to the increase of free radical production and decreased scavenging capacity, which leads to the accumulation of ROS in vivo and caused oxidative damage. Many studies have shown that the invasion of pathogens in organisms increases the level of ROS and changes redox status, affecting the function of the antioxidant system and gene expression^44,45^. The harmful effect of pathogens infection on free radical scavenging ability was partly attributed to the reduction of antioxidant enzymes such as T-SOD, CAT, GPX, GST, GR and GSH content^46^. The level of ROS increased after WSSV infected, which caused oxidative stress. Compared with uninfected animals, the activities of SOD, CAT, GST, GPx, GR and the content of GSH decreased significantly in WSSV infected animals^47^. In this study, we found that the level of ROS, MDA and PC in infected turtles were significantly decreased by treatment with NAC, and the muscle antioxidant enzyme activities increased (Fig. 2). In a word, the improvement of antioxidant enzyme activity may be related to the improvement of the ability of scavenging reactive oxygen species^48^.

In addition, the expression of antioxidant genes (except GPx1 and GSTZ1) were significantly decreased in the muscle of turtles at the molecular level (Fig. 3), which was consistent with the results of enzyme activity. This indicates that the regulation of transcription level may be play an important role in the oxidative stress of turtles. Nrf2 is an important transcription factor regulating the redox balance of cells, and Keap1/Nrf2 is an important antioxidant signaling pathway in maintaining the balance between antioxidants and peroxides in vivo. Nrf2 combines with the antioxidant response elements (ARE) in the upstream promoter region of these antioxidant genes, and promotes the transcription of antioxidant genes including SOD, CAT, GPX and GST^49^. Our results showed that down-regulated expression of Nrf2 signaling molecule and up-regulated levels of Keap1 mRNA in the muscle of turtles after infection (Fig. 3). Meanwhile, the expression trend of antioxidant enzyme genes was consistent with that of Nrf2 mRNA, but opposite to that of Keap1 after the pathogenic bacteria invaded turtles. It was suggested that the expression level of antioxidant enzyme genes in infected turtles may be due to the regulated by Keap1/Nrf2 signaling pathway, and its mechanism of action remains to be further studied. On the contrary, promoting the translocation of Nrf2 to the nucleus also plays a key role in up-regulating the expression of antioxidant enzyme gene in aquatic animals. Studies have shown that up-regulated expression of Nrf2 could increase SOD, CAT, GPX and GST mRNA levels in the muscle^50^. Keap1 binds to Nrf2 directly in the cytoplasm, which makes Nrf2 ubiquitinate and degrade by proteasome, thus inhibiting the nuclear translocation of Nrf2^49^. However, the down-regulating Keap1 results in Nrf2 nuclear translocation, thus up-regulated the expression of downstream antioxidant genes. In addition, the signal molecule Keap1 was significantly up-regulated, suggesting that the pathogenic bacteria could inhibit Nrf2 transport to the nucleus by up-regulating the expression of Keap1 in the muscle, and thereby decreasing the expression of antioxidant enzyme genes^51^. In this study, after the injection of NAC oxidative stress levels were significantly reduced in the pathogen infection group and the mRNA levels of SOD, CAT, GSTCD, and GSTO1 increased significantly (Fig. 3). At present, studies have shown that the antioxidant nanoparticles have potential improvement potential in controlling the cytotoxicity of *P. aeruginosa* and pyocyanin^52^. Moreover, Shay et al. (2012) founded that upstream signaling molecules can regulate Nrf2 expression, such as mTOR^53^. The decreased expression of mTOR can down-regulated the expression of Nrf2 in endothelial cells of brain^54^. In this study, the expression of signaling molecule mTOR was significantly down-regulated after infection with pathogens in the muscle (Fig. 4). The result of correlation analysis indicated that Nrf2 was positively correlated with mTOR. This suggests that the down-regulating Nrf2 might be related to mTOR down-regulated after pathogens infected.

ROS can also induce autophagy activation by a variety of mechanisms. Autophagy is an evolutionarily highly conserved catabolic process, which plays an important role in cell growth and development, homeostasis and remodeling, thus helping to maintain the balance between cell component degradation, synthesis and recycling^55^. Microtubule-associated protein 1 light chain 3 (MAP1LC3) is mainly located on the membrane of autophagy, which is involved in the formation of autophagy and serves as the main autophagy marker. The MAP1LC3 family consists of three highly homologous members, MAP1LC3A, MAP1LC3B and MAP1LC3C^56,57^. Our study showed that the expression of autophagy-related genes ULK1, ATG13, ATG101, Beclin-1, ATG14, MAP1LC3A, and MAP1LC3B was significantly increased and that SQSTM1 was decreased in the muscle with pathogen infection. These observations demonstrate that *P. vulgaris* and *E. meningoseptica* infection respond to ROS and promote autophagy in muscle of turtles. This indicates that ROS might activate both transcriptional and nontranscriptional mechanisms that initiate autophagy in turtles. Numerous studies have shown that many conditions can cause an increase of ROS level, and the accumulation of autophagy marker protein LC3 can be detected. In some diseases, autophagy is caused by increased ROS level. Such as tumor necrosis factor α (TNFα) caused the mitochondrial dysfunction significantly and produces a large number of ROS, which induce mitochondrial autophagy during necrotizing enterocolitis^58,59^. Moreover, we observed that increases in partial autophagy-related genes expression after infection were eliminated by the antioxidant NAC. The expression levels of ULK1, MAP1LC3A and MAP1LC3B were significantly decreased after treatment with NAC. This suggests that NAC may be inhibited the occurrence of autophagy through antioxidant effect. We further demonstrated that the expression of LC3 protein was significantly increased by pathogen infection, and then treatment with NAC was reduced significantly but did not return to normal levels (Fig. 5A). Furthermore, the TEM results showed that there were obviously more autophagosomes in muscle with infectious pathogens, however, there was a decrease after treatment with NAC (Fig. 5B). Finally, we also found that the expression of mTOR mRNA level decreased significantly, and increased AMPK mRNA level in the pathogen infection group (Fig. 4). Rapamycin target protein (mTOR) has been proved to be a negative regulatory factor of autophagy, which responds to the influence signals from nutrients and growth factor pathway, and then can control cell growth and metabolism^60^. In contrast, AMPK positively regulates autophagy. AMPK is activated by the pressure of energy change, which can be sensed by the change of AMP/ATP ratio. After activation, AMPK stimulates autophagy through various mechanisms. This is because it can promote an early reaction induced by autophagy by phosphorylation and activation of ULK1 and Beclin-1-VPS34 complexes. Secondly, AMPK inhibits mTOR by phosphorylation^61,62^. Our study shows that the infection pathogens could significantly increase the expression of AMPK and decreased mTOR as compared to NC group. After NAC injection, mTOR was up-regulated and AMPK down-regulated compared with infection group (Fig. 4). This suggests that ROS may mediate the molecular mechanism of AMPK-mTOR pathway to induce autophagy formation.

## Conclusion

In summary, the present study indicated that muscle nutritional characteristics were dramatically changed after pathogen infection and increased the level of ROS caused by a change in the redox state, which induced oxidative stress and autophagy in the muscle of Chinese soft-shelled turtles. However, N-acetylcysteine treatment could ameliorate the process perhaps by decreasing the ROS level and regulating Nrf2-antioxidant signaling pathway. These results provide a biochemical and molecular basis for the effects of bacterial infection on aquatic animals, at least Chinese soft-shelled turtles and may put forward its potential significance in muscle nutritional quality, health and disease control.

## Supplementary Information

Supplementary Information.

## Data Availability

All data relevant to the study are included in the paper or uploaded as supplementary information. No additional data are available.
