# Supplementary material for: Investigation of Proteus vulgaris and Elizabethkingia meningoseptica invasion on muscle oxidative stress and autophagy in Chinese soft-shelled turtle (Pelodiscus sinensis)
Source: Sci Rep. 2021 Feb 11;11:3657. doi: 10.1038/s41598-021-83388-6 (PMC7878920; doi:10.1038/s41598-021-83388-6)
Supplement: Supplementary file 1 — Supplementary Information. [file 41598_2021_83388_MOESM1_ESM.pdf]

Title page

Investigation of *Proteus vulgaris* and *Elizabethkingia meningoseptica* invasion on muscle oxidative stress and autophagy in Chinese soft-shelled turtle (*Pelodiscus sinensis*)

Hong-Hui Li[1,2], Ling-Sheng Bao[1], Shi-Ming Deng[3], Li Liu[3], Jia Cheng[1], Xiao Chen[1], Ya-Xiong Pan[1], Jian-She Zhang[1] ✉ & Wu-Ying Chu[1] ✉

[1]Hunan Provincial Key Laboratory of Nutrition and Quality Control of Aquatic Animals, College of Biological and Environmental Engineering, Changsha University, Changsha, China. [2]College of Chemistry and Food Engineering, Changsha University of Science & Technology, Changsha, China. [3]Hunan Fisheries Science Institute, Changsha, China. ✉ e-mail: jzhang@ccsu.cn; chuwuying18@163.com

Correspondence to jzhang@ccsu.cn; chuwuying18@163.com

Supplementary figure

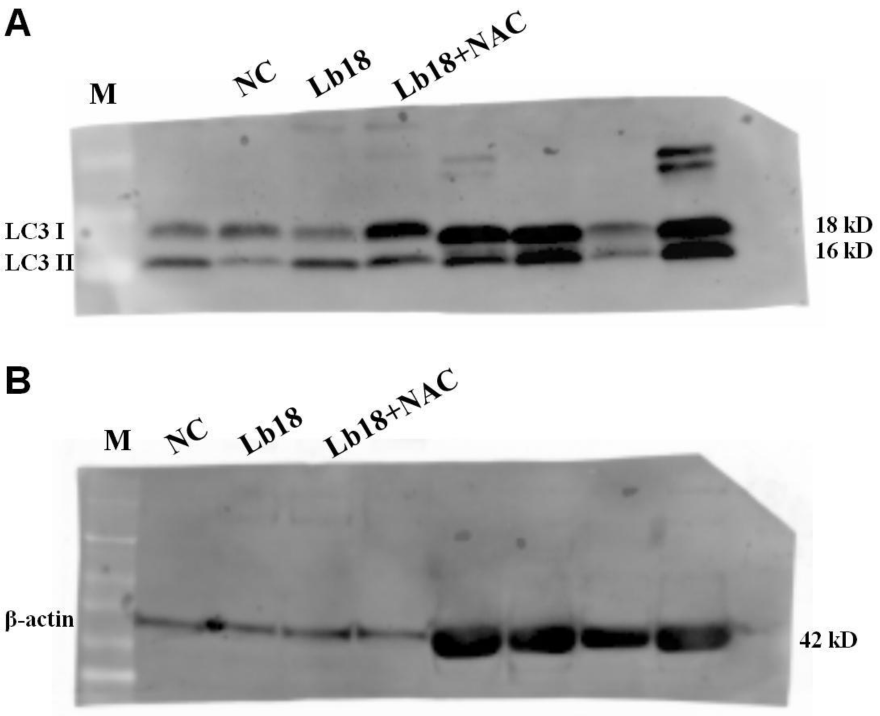

Suppl Figure. Full-length blots are presented in Supplementary Figure 5A. Autophagy detection using Western blot, and the NIH ImageJ software was used for quantitative analysis of protein bands. (A) Western blot analysis of LC3 protein, LC3 I and LC3 II. (B) Western blot analysis of β-actin protein.
